# rno-miR-128-3p promotes apoptosis in rat granulosa cells (GCs) induced by norepinephrine through Wilms tumor 1 (WT1)

**DOI:** 10.1007/s11626-021-00609-y

**Published:** 2021-09-23

**Authors:** Ming Li, Ling Xue, Weibin Xu, Pingping Liu, Feng Li

**Affiliations:** 1 Department of Pharmacy, Lian Yungang Hospital of Traditional Chinese Medicine, Affiliated Hospital of Nanjing University of Chinese Medicine, Jiang Su Province, Lianyungang, 222000 People’s Republic of China; 2 Pharmacy Department, Shandong Qingdao Hospital of Integrated Traditional Chinese and Western Medicine, No. 3 Jiaxiang Road, South District, Qingdao City, Shandong Province 266002 China; 3 Pharmacy Department, Gaoqing County People’s Hospital, No.11 Qingcheng Road, Gaoqing County, Zibo City, 256300 Shandong Province China; 4 Pharmacy Department, Liaocheng Chiping District People’s Hospital, No. 1057 Culture Road, Chiping County, Liaocheng City, 252100 Shandong Province China; 5 Drug Dispensing Department, Zibo Central Hospital, No. 54 The Communist Youth League West Road, Zhangdian District, Zibo City, 255000 Shandong Province China

**Keywords:** Norepinephrine, Rno-miR-128-3p, WT1, Granulosa cell

## Abstract

The mechanism related to ovarian follicular is complex, which has not been fully elucidated. Abundant reports have confirmed that the ovarian function development is closely related to sympathetic innervation. As one of the major neurotransmitters, norepinephrine (NE) is considered an effective regulator of ovarian functions like granulosa cell (GC) apoptosis. However, the mechanism between NE and GC apoptosis in rat is still unclear. In our study, GCs were isolated and cultured in vitro with NE treatment. The apoptosis of GCs was facilitated by NE. Wilms tumor 1 (WT1) was found to be significantly downregulated in GCs after NE treatment, and overexpression of WT1 repressed apoptosis in rat GCs induced by NE. rno-miR-128-3p was found to be significantly enhanced by NE in GCs, and inhibition of rno-miR-128-3p repressed apoptosis in rat GCs induced by NE. Mechanistically, rno-miR-128-3p interacted with WT1 and repressed its expression. In summary, inhibition of rno-miR-128-3p may enhance WT1 expression, and then repress NE-induced apoptosis in rat GCs. Our research may provide a new insight for the improvement of ovarian follicular development.

## Introduction

Follicle development is a complex physiological process that is crucial to ovarian function and fertility (Fortune 1994). When the follicles begin to develop, the static follicles are activated and gradually result in the growth and selection of dominant follicles (Murray and Spears 2000; Webb 2003). Interruption of follicle development may lead to an increase in follicular atresia, which is a main reason for ovarian pathology (Voorhis 2008). Hence, investigating the potential mechanisms of follicle development is crucial for the treatment of ovarian disease.

As one of the major neurotransmitters, norepinephrine (NE) is considered an effective regulator of ovarian functions like granulosa cell (GC) apoptosis (Zhang *et al*. 2017a). There are two pathways to the sympathetic innervation in the rodent ovaries, including the upper nerves of the ovary extending along the ovary ligaments and the plexus nerves mainly related to the vasculature (Campo 2019). NE level plays a crucial role in regulating ovarian cystic health. In ovarian cancer, NE stimulated cell invasion by elevating hTERT expression (Choi 2015). A study conducted by Patel *et al.* (2015) found that NE decreases ROS and DNA injury in ovarian surface epithelial cells. NE is reported to be related with the ROS production in GCs, which acts as important factors in ovarian physiology, like ovulation (Saller 2012). However, it is not clear whether and how NE affects the GC apoptosis in rat.

MicroRNA (miR/miRNA) is a small RNA that can directly bind to the 3′UTR of targeted mRNA, as a result of mRNA degradation. miRNA represses gene transcription of target (Hu 2018). Vast evidences revealed that miRNAs played pivotal roles in ovarian disease, like polycystic ovary syndrome (PCOS) (Huang 2020) and ovarian cancer (Feng *et al*. 2020). A study conducted by Wang *et al.* (2020) found that miR-29 regulates GC function through suppressing PTX3. And miR-130b is involved in the development of PCOS through regulating Cx43 (Jiang *et al*. 2020). miR-128-3p is reported to be elevated in GCs of diminished ovarian reserve (Woo 2018), but whether and how miR-128-3p affects the GC apoptosis was unknown.

The Wilms tumor gene 1 (WT1) encodes a zinc finger transcription factor in gonads, including testis and ovary (Pelletier 1991; Chun 1999). WT1 played crucial effects on the gonad development. In the ovaries, WT1 inhibition results in cell ectopic development, which in turn decreases ovary weight and the number of large sinus-shaped follicles (Gao 2014; Chen 2017). Besides, WT1 alters GC apoptosis and proliferation by regulating the wnt/β-catenin pathway (Wang *et al*. 2019). The expression of WT1 is regulated by miRNAs. For example, miR-361-5p represses the development of hepatocellular carcinoma by repressing WT1 (Cheng 2019). Magnesium isoglycyrrhizinate diminishes fructose-induced apoptosis of podocytes via decrease of miR-193a to upregulate WT1 (Li 2019a). The bioinformatics analysis predicted that miR-128-3p was a candidate to upregulate WT1. However, there was no relevant report on miR-128-3p and WT1. So we focus on microRNA and WT1 in the mechanism of norepinephrine-inducing apoptosis.

In this research, we aimed to investigate the effects of miR-128-3p on rat GCs. This study used GCs isolated from rat ovary as a cell model to evaluate the function of miR-128-3p on GCs, and the underlying mechanism was illuminated by evaluating the relation between miR-128-3p and WT1. Our findings may offer a new insight for the improvement of ovarian follicular development.

## Materials and methods

### Ethical approval

Sprague–Dawley (SD) rats (age 21 d; weighing 45–55 g) were acquired from Shanghai Slyke Experimental Animals Co., Ltd. (Shanghai, China). Rats were treated humanely using approved procedures granted by the local Animal Ethics Committee.

### GC culture and treatment

Ovaries were collected and put in serum-free high-glucose Dulbecco’s Modified Eagle’s Medium (DMEM), followed by washing and suspending in DMEM with 5% fetal bovine serum (FBS). After puncturing the follicle with the fine needles, the GCs are strongly precipitated and washed by using the medium. GCs were collected and cultured in DMEM with 10% FBS (Shen and Wang 2019). The primary GCs were transfected after 48-h culture.

For NE treatment, NE is dissolved in DMSO; GCs were treated with 0, 0.1, 1, and 10 μmol/L NE for 24 h, or treated with 10 μmol/L NE for 0, 6, 12, 24, and 48 h, respectively. The control experiment is all added with the DMSO vehicle for transfection; GCs were transfected for 6 h before NE treatment (Zhang *et al*. 2017b). Lipofectamine™ 3000 (Invitrogen, Thermo Fisher, Carlsbad, CA) was utilized. PcDNA-WT1, rno-miR-128-3p mimics, rno-miR-128-3p inhibitor, si-WT1, and negative control (NC) were obtained from Genepharma (Shanghai, China).

### TUNEL assay

Twenty-four hours after NE treatment, GCs were fixed using 2% formalin at 37°C for 1 h, using 0.1% Triton X-100 to permeate on ice for 2 min, followed by 1-h incubation with 50 μL TUNEL reaction mixture at 37°C under dark conditions. The results were observed under fluorescence microscope (LSM880, Zeiss, Oberkochen, Germany) (Li 2019b).

### Flow cytometry assay

1.00 × 10^6^ cells/mL was applied for PI staining for 10 min and subjected to cell apoptosis analysis using FITC and FACSCalibur flow cytometer (342,975, BD, Franklin Lakes, NJ). The number of cells was acquired at 488-nm wavelength and analyzed by Cell Quest software.

### RT-qPCR assay

Total RNA in GCs was obtained by TRIzol (Sigma-Aldrich, St. Louis, St. Louis, MO) following the instructions. RT-PCR was carried by employing TaqMan™ MicroRNA Reverse Transcription Kit (Applied Biosystems™, Foster City, CA) for rno-miR-128-3p and RevertAid RT Reverse Transcription Kit (Thermo Scientific™, CA) for genes; qRT-PCR was performed as previously described (Wang 2019). The primers (Zhang *et al*. 2017b; Li 2019c; Zhang 2019, 2020; Harishkumar and Selvaraj 2020; Wu 2020) are listed in Table 1. U6 and GAPDH served as an endogenous control.

**Table 1:** Primer sequences for qRT-PCR

Primer name	(5′–3′) primer sequences
Caspase-3-forward	5′-ATGTCGATGCAGCTAACCTC-3′
Caspase-3-reverse	5′-TCCTTTTGCTGTGATCTTCC-3′
Caspase-9-forward	5′-CTGAGCCAGATGCTGTCCCATA-3′
Caspase-9-reverse	5′-GACACCATCCAAGGTCTCGATGTA-3′
BAX-forward	5′-TTTCATCCAGGATCGAGCAGG-3′
BAX-reverse	5′-GCAAAGTAGAAGAGGGCAACCAC-3′
BCL2-forward	5′-CTACCGTCGTGACTTCGCA-3′
BCL2-reverse	5′-TACCCAGCCTCCGTTATCC-3′
WT1-forward	5′-GTGGCTCCTGCGTTTCCCCC-3′
WT1-reverse	5′-CCACAGGCATG GCGCGG-3′
GAPDH-forward	5′-ATGGCAGACGATGATCCCTAC-3′
GAPDH-reverse	5′-CGGAATCGAAATCCCCTCTGTT-3′
rno-miR-128-3p-forward	5′-GGTCACAGTGAACCGGTC-3′
rno-miR-128-3p-reverse	5′-GTGCAGGGTCCGAGGT-3′
U6-forward	5′-CTCGCTTCGGCAGCACA-3′
U6-reverse	5′-AACGCTTCACGAATTTGCGT-3′

#### Western blot

Cell lysate was isolated utilizing cell lysis buffer (Beyotime, Nanjing, China). Western blots were conducted accordingly as previously described (Chowdhury 2013). Briefly, the membrane was transferred and blocked at 37°C for 2 h, followed by primary antibody incubation: rabbit anti-caspase-3 (1:500, ab13847), anti-cleaved caspase-3 (1:5000, ab49822), anti-caspase-9 (1:1000; ab184786), anti-cleaved caspase-9 (1:2000, ab2324), anti-BAX (1:5000; ab32503), anti-BCL2 (1:1000, ab59348), anti-WT1 (1:100, ab224806), and anti-β-actin (1:5000, ab179467), and then re-probed with IgG complexed to horseradish peroxidase (1:2000, ab6721) antibody. The chemiluminescence kit (Millipore, Darmstadt, Germany) was adopted to observe the immune response zone, and ImageJ software (ImageJ Software Inc., MD) was used to quantify the integrated density of each band. Antibodies mentioned before were supplied by Abcam (Cambridge, UK).

#### Dual luciferase reporter assay

In order to confirm that WT1 was a target of rno-miR-128-3p, WT1-WT (containing the binding sites of rno-miR-128-3p at WT1 3′UTR) and WT1-MUT (mutation of binding sites) were cloned into Luciferase Reporter Vector (AM5795, Invitrogen, Carlsbad,, CA). WT1-WT or WT1-MUT were co-transfected with rno-miR-128-3p mimics into GCs. Forty-eight hours after transfection, GCs were explored using a Reporter Vector System (AM5795, Invitrogen, CA) via a Glomax20/20 luminometer (Promega, Madison, WI) (Zhang *et al*. 2017b).

#### Statistical analysis

The mean ± SD represented data from three independent experiments. SPSS 21.0 (IBM Corp., Armonk, NY) was applied for statistical analysis of all data. Student’s *t* test was used for comparison between two groups, and one-way ANOVA and Tukey’s posttests were used for multiple groups. The level of significance was *P* < 0.05.

## Results

### NE induces apoptosis in rat GCs and represses the expression levels of WT1

**Figure 1.**
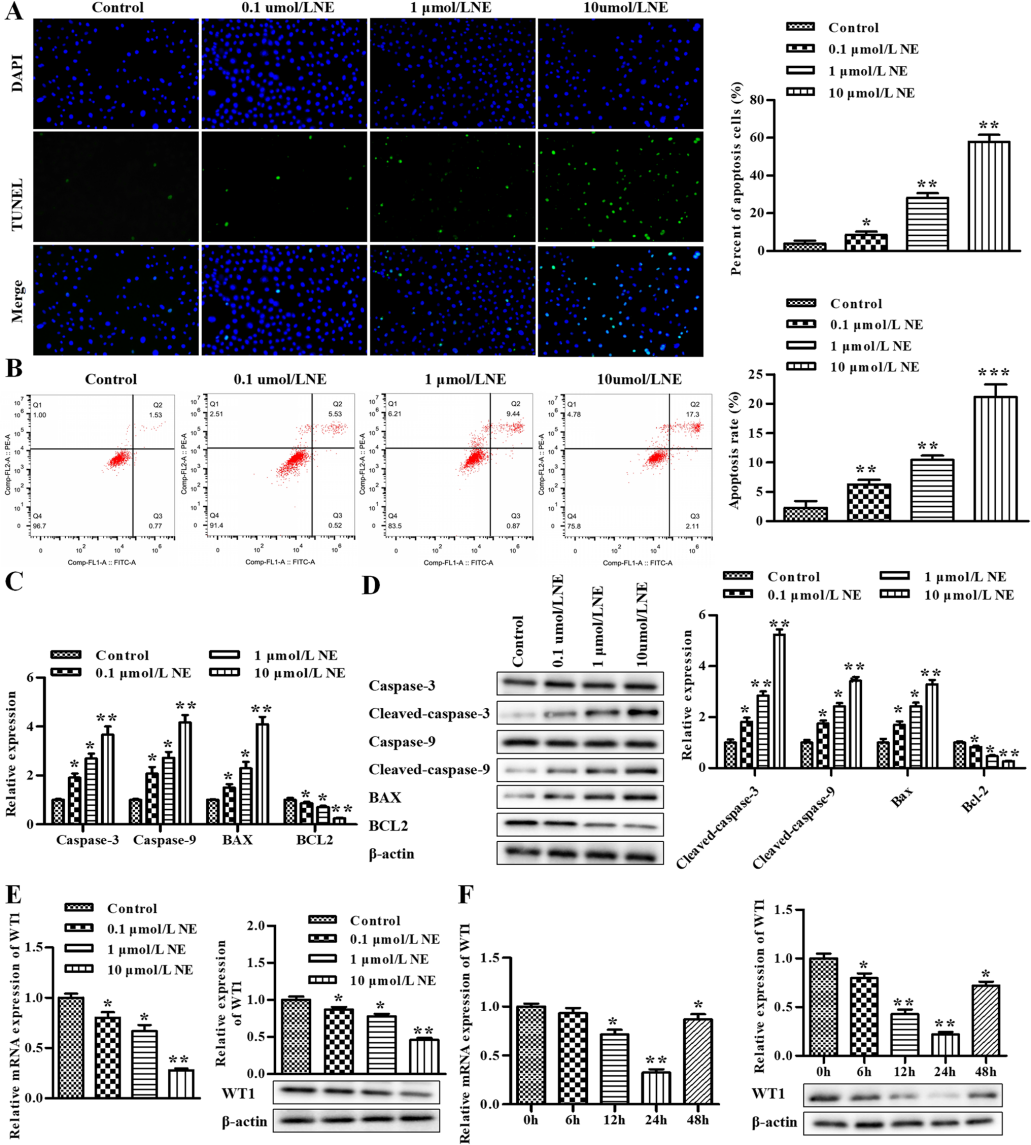
NE induces apoptosis in rat GCs and represses the expression levels of WT1. (*A*) The cell apoptosis was determined using TUNEL assay in rat GCs. (*B*) The cell apoptosis was determined using flow cytometry assay*. *(*C*) The levels of caspase-3, caspase-9, BAX, and BCL2 were determined by qRT-PCR. (*D*) The levels of caspase-3, cleaved caspase-3, caspase-9, cleaved caspase-9, BAX, and BCL2 were determined by Western blot assay. (*E*) The levels of WT1 was determined by qRT-PCR and Western blot after treating with different concentrations of NE. (*F*) The levels of WT1 was determined by qRT-PCR and Western blot after treating with 10 μmol/L NE for 0, 6, 12, 24, and 48 h, respectively. *P < 0.05, **P < 0.01 vs. control group. Data is shown as mean ± SD (*n* = 3).

After treating with different concentrations of NE, the apoptosis of GCs was assessed by the TUNEL assay and flow cytometry assay. As shown in Fig. 1*A* and *B*, the apoptosis was elevated in a NE concentration-dependent manner. RT-qPCR was conducted to assess the levels of caspase-3, caspase-9, BAX, and BCL2, and our data exhibited that the mRNA of caspase-3, caspase-9, and BAX was significantly improved by NE in a concentration-dependent manner, while the results of BCL2 were the opposite (Fig. 1*C*). What’s more, the same phenomenon was observed via the Western blot assay, which exhibited that the protein of cleaved caspase-3, cleaved caspase-9, and BAX was significantly improved by NE in a concentration-dependent manner, while the results of BCL2 were the opposite (Fig. 1*D*).

Next, we further verified the effect of NE on the level of WT1. After being treated with different concentrations of NE, RT-qPCR and Western blot assay were conducted to assess the level of WT1. Our data exhibited that both mRNA and protein of WT1 were decreased by NE treatment and showed NE concentration dependence (Fig. 1*E*); then, 10 μmol/L NE was selected for subsequent experiments. Furthermore, GCs were treated with 10 μmol/L NE for 0, 6, 12, 24, and 48 h, respectively. The results of RT-qPCR and Western blot assay exhibited that the level of WT1 was lowest for the time of 24 h. As a result, 24-h treatment was selected for subsequent experiments.

### Overexpression of WT1 represses apoptosis in NE-treated rat GCs

**Figure 2.**
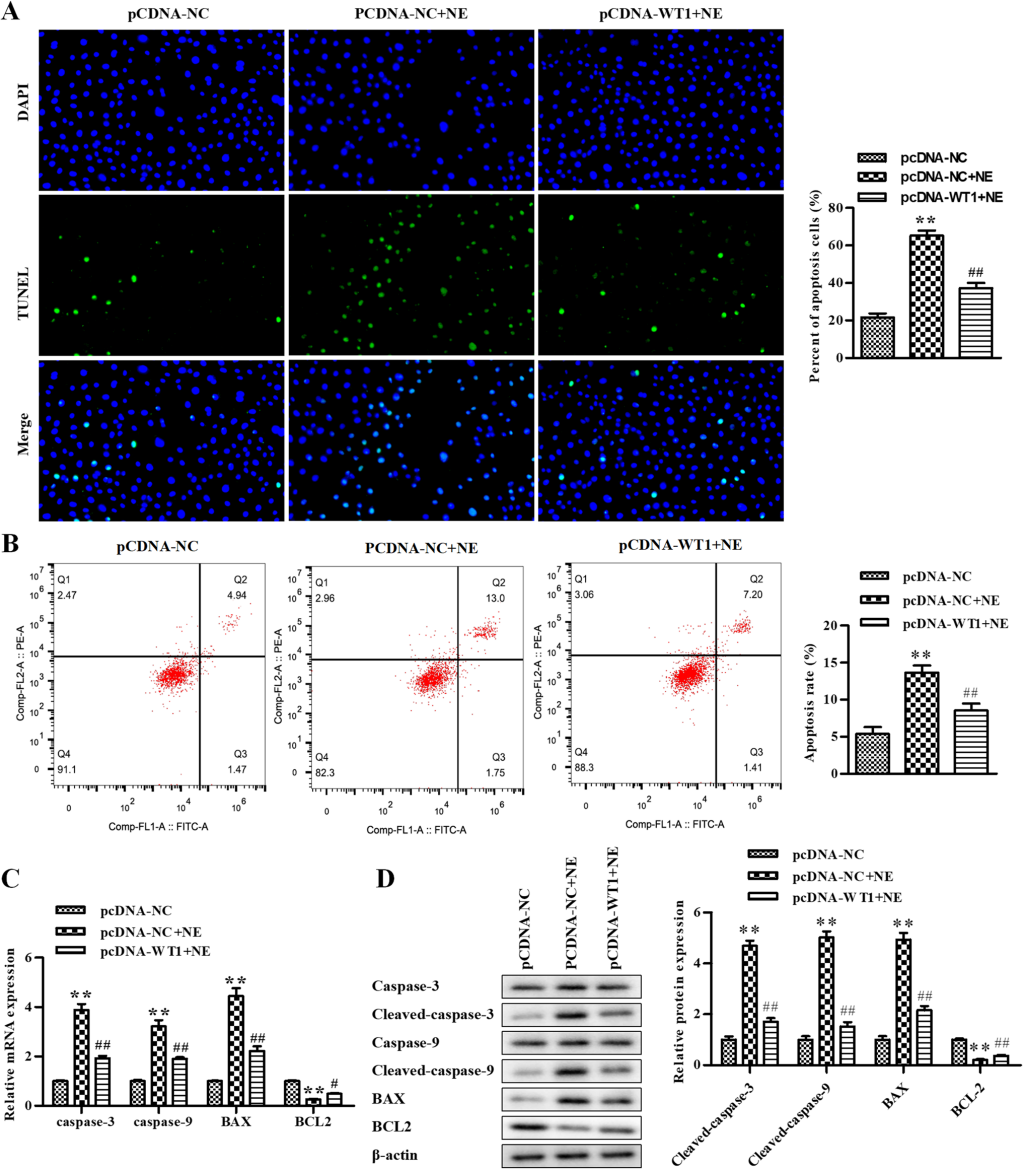
Overexpression of WT1 represses apoptosis in NE-treated rat GCs. (*A*) The cell apoptosis was determined using TUNEL assay in rat GCs. (*B*) The cell apoptosis was determined using flow cytometry assay. (*C*) The levels of caspase-3, caspase-9, BAX, and BCL2 were determined by qRT-PCR. (*D*) The levels of caspase-3, cleaved caspase-3, caspase-9, cleaved caspase-9, BAX, and BCL2 were determined by Western blot assay. *P < 0.05, **P < 0.01 vs. pcDNA-NC. Data is shown as mean ± SD (*n* = 3).

After transfecting NE-treated GCs with pcDNA-WT1 to upregulate WT1 expression, the TUNEL assay and flow cytometry assay were conducted to assess the apoptosis of GCs. The data revealed that NE elevated GC apoptosis compared with the pcDNA-NC group, which was alleviated by pcDNA-WT1 (Fig. 2*A* and *B*). RT-qPCR was conducted to assess the levels of caspase-3, caspase-9, BAX, and BCL2, and our data exhibited that the mRNA of caspase-3, caspase-9, and BAX was significantly improved after being treated with NE, whereas it was reversed by pcDNA-WT1 and the results of BCL2 were the opposite (Fig. 2*C*). What’s more, similar results were observed via the Western blot assay, which exhibited that the protein of cleaved caspase-3, cleaved caspase-9, and BAX was significantly improved by NE, whereas it was reversed by pcDNA-WT1and the results of BCL2 were the opposite (Fig. 2*D*). Taken together, GC apoptosis induced by NE was repressed due to WT1 overexpression.

### WT1 is a target gene of rno-miR-128-3p

**Figure 3.**
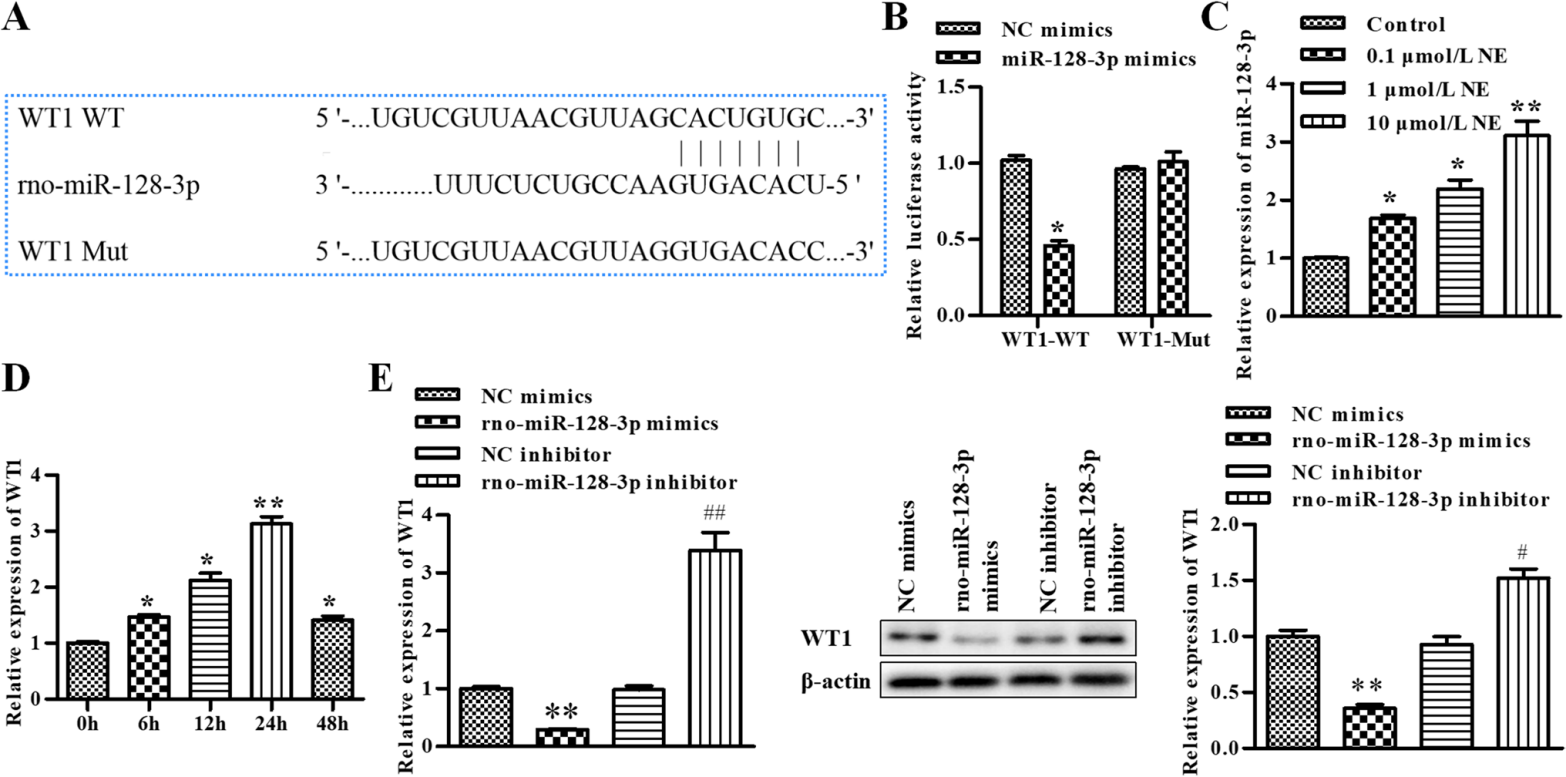
WT1 is a target gene of rno-miR-128-3p. (*A*) WT1 was predicted as a target of rno-miR-128-3p via bioinformatics analysis. (*B*) rno-miR-128-3p was bound to 3′UTR of WT1 using dual luciferase reporter assay, *P < 0.05, **P < 0.01 vs. NC mimics. (*C*) The levels of rno-miR-128-3p was determined by qRT-PCR after treating with different concentrations of NE, *P < 0.05, **P < 0.01 vs. control. (*D*) The levels of rno-miR-128-3p was determined by qRT-PCR after treating with 10 μmol/L NE for 0, 6, 12, 24, and 48 h, respectively, *P < 0.05, **P < 0.01 vs. 0 h. (*E*) The level of WT1 was determined by qRT-PCR and Western blot, *P < 0.05, **P < 0.01 vs. NC mimics or NC inhibitor. Data is shown as mean ± SD (*n* = 3).

WT1 was predicted as a target of rno-miR-128-3p via bioinformatics analysis (Fig. 3*A*), which was further verified by dual luciferase reporter assay. We co-transfected rno-miR-128-3p mimics and WT1-WT or WT1-Mut into GCs, and the data revealed that rno-miR-128-3p mimics markedly suppressed the luciferase activity of WT1-WT (Fig. 3*B*). Treated with different concentrations of NE, RT-qPCR was conducted to assess the level of rno-miR-128-3p. Our data exhibited that the level of rno-miR-128-3p was enhanced by NE and showed NE concentration dependence (Fig. 3*C*); then, 10 μmol/L NE was selected for subsequent experiments. Furthermore, GCs were treated with 10 μmol/L NE for 0, 6, 12, 24, and 48 h, respectively. The results of RT-qPCR assay exhibited that the level of rno-miR-128-3p was highest for the time of 24 h. As a result, 24-h treatment was selected for subsequent experiments (Fig. 3*D*).

To further verify the effect of rno-miR-128-3p on the level of WT1, we transfected GCs with rno-miR-128-3p mimics and rno-miR-128-3p inhibitor; RT-qPCR and Western blot assay were conducted to assess the level of WT1. Our data exhibited that both mRNA and protein of WT1 were repressed by rno-miR-128-3p mimics and elevated by rno-miR-128-3p inhibitor (Fig. 3*E*). Taken together, these results supported the idea that WT1 was a putative target of rno-miR-128-3p.

### Inhibition of rno-miR-128-3p represses apoptosis in rat GCs treated with NE

**Figure 4.**
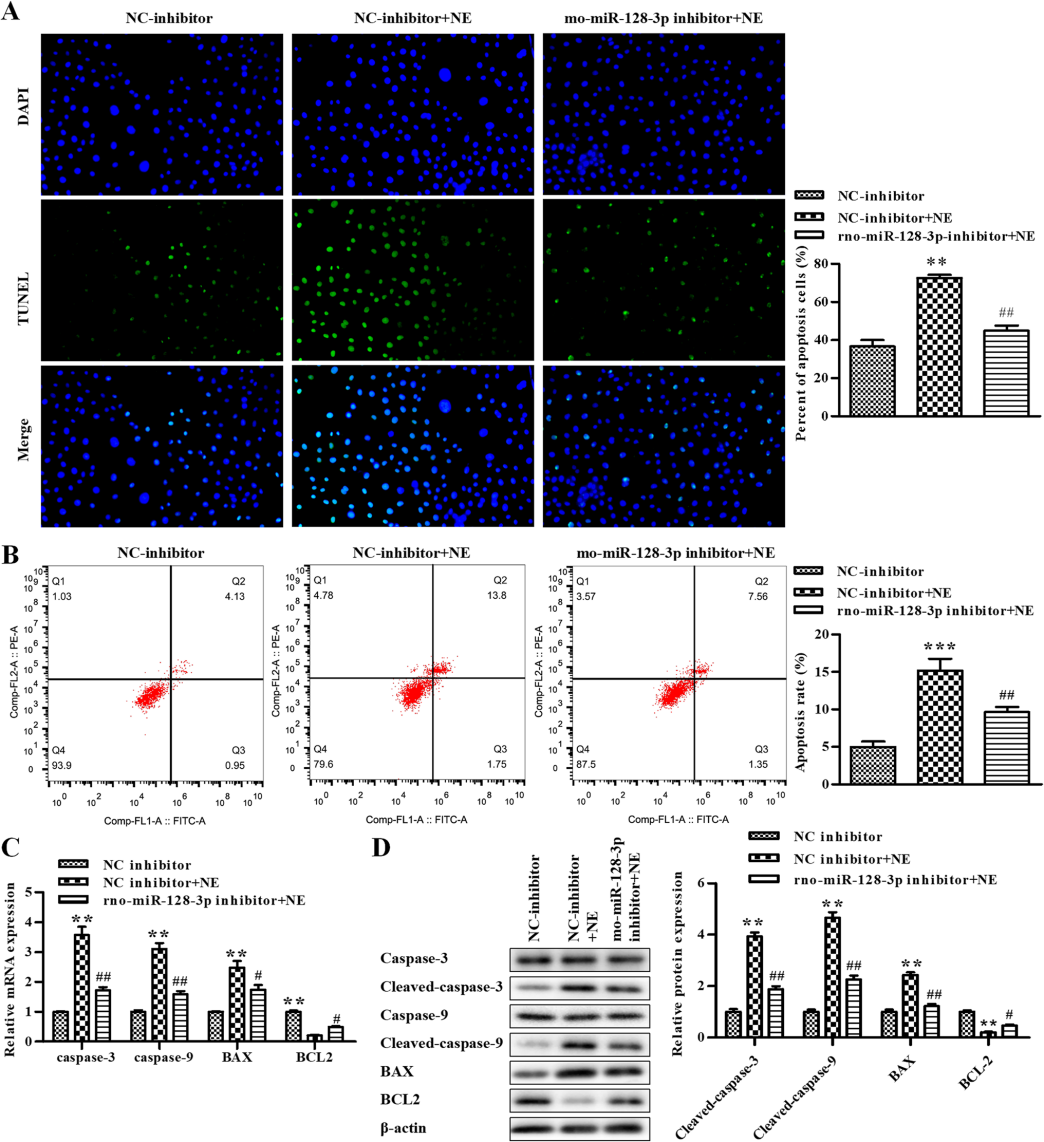
Inhibition of rno-miR-128-3p represses apoptosis in rat GCs treated with NE. (*A*) The cell apoptosis was determined using TUNEL assay in rat GCs. (*B*) The cell apoptosis was determined using flow cytometry assay. (*C*) The levels of caspase-3, caspase-9, BAX, and BCL2 were determined by qRT-PCR. (*D*) The levels of caspase-3, cleaved caspase-3, caspase-9, cleaved caspase-9, BAX, and BCL2 were determined by Western blot assay. *P < 0.05, **P < 0.01 vs. NC inhibitor. Data is shown as mean ± SD (*n* = 3).

To further verify the effect of rno-miR-128-3p on GC apoptosis, NE-treated GCs were transfected with rno-miR-128-3p inhibitor, and the TUNEL assay and flow cytometry assay were conducted to assess the apoptosis of GCs. The data revealed that NE elevated GC apoptosis compared with the NC inhibitor group, which was alleviated by rno-miR-128-3p inhibitor (Fig. 4*A* and *B*). RT-qPCR was conducted to assess the levels of caspase-3, caspase-9, BAX, and BCL2, and our data exhibited that the mRNA of caspase-3, caspase-9, and BAX was significantly improved after being treated with NE, whereas it was reversed by rno-miR-128-3p inhibitor, while the results of BCL2 were the opposite (Fig. 4*C*). The data of the Western blot assay exhibited that the protein of cleaved caspase-3, cleaved caspase-9, and BAX were significantly improved by NE, whereas it was reversed by rno-miR-128-3p inhibitor and the results of BCL2 were the opposite (Fig. 4*D*). Taken together, GC apoptosis induced by NE was repressed by rno-miR-128-3p inhibitor.

### WT1 mediates the effects of rno-miR-128-3p on NE-treated rat GCs

**Figure 5.**
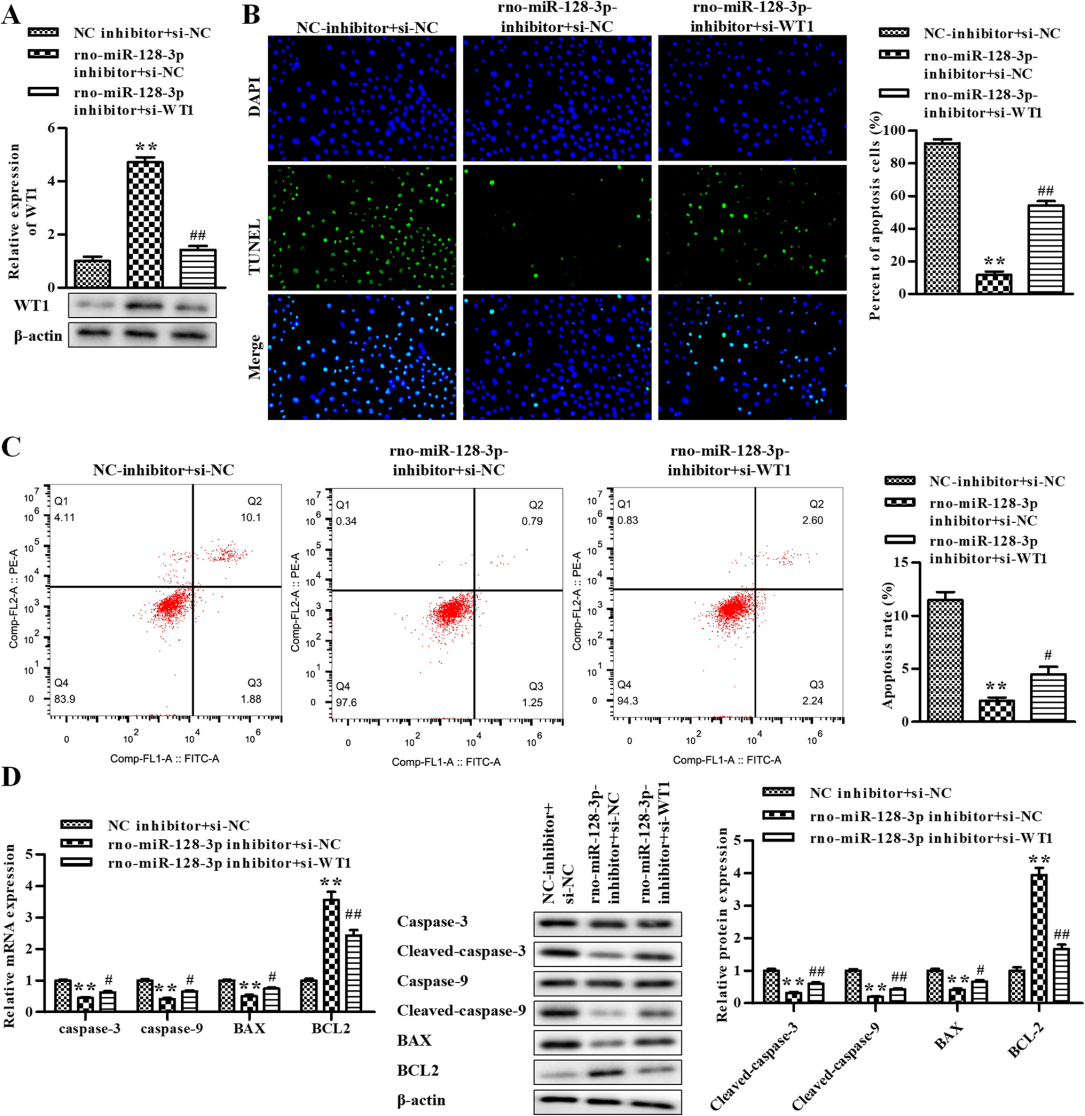
Inhibition of rno-miR-128-3p represses NE-induced apoptosis in rat GCs by WT1. (*A*) The level of WT1 was determined by Western blot. (*B*) The cell apoptosis was determined using TUNEL assay in rat GCs. (*C*) The cell apoptosis was determined using flow cytometry assay. (*D*) The levels of caspase-3, caspase-9, BAX, and BCL2 were determined by qRT-PCR. (*E*) The levels of caspase-3, cleaved caspase-3, caspase-9, cleaved caspase-9, BAX, and BCL2 were determined by Western blot assay. *P < 0.05, **P < 0.01 vs. NC inhibitor + si-NC. Data is shown as mean ± SD (*n* = 3).

In order to evaluate the functions of rno-miR-128-3p and WT1 in GC apoptosis induced by NE, we transfected rno-miR-128-3p inhibitor and si-WT1 into NE-treated GCs at the same time, followed by the Western blot assay. The data of Western blot assay exhibited that the protein of WT1 was observably stimulated by rno-miR-128-3p inhibitor, which was attenuated by si-WT1 (Fig. 5*A*). Next, the apoptosis of GCs was assessed by the TUNEL assay and flow cytometry assay. As shown in Fig. 5*B* and *C*, the apoptosis was significantly reduced by rno-miR-128-3p inhibitor, whereas it was reversed by si-WT1. RT-qPCR was conducted to assess the levels of caspase-3, caspase-9, BAX, and BCL2, exhibiting that the mRNA of caspase-3, caspase-9, and BAX was significantly diminished by rno-miR-128-3p inhibitor, whereas it was reversed by si-WT1 and the results of BCL2 were the opposite (Fig. 5*D*). The data of the Western blot assay exhibited that the protein of cleaved caspase-3, cleaved caspase-9, and BAX was significantly reduced by rno-miR-128-3p inhibitor, whereas it was reversed by si-WT1 and the results of BCL2 were the opposite (Fig. 5*E*). Taken together, inhibition of rno-miR-128-3p repressed NE-induced apoptosis in rat GCs by elevating WT1.

## Discussion

Studies have proved that NE is related to various aspects of ovarian function, like early follicle development (Mayerhofer 1997), its association with the steroidogenesis, and apoptosis on dioestrus II of the ovary in rats (Bronzi *et al*. 2015). Recent studies indicate that NE serves as beta-2 epinephrine receptors in rat GCs and promotes progesterone level, but it does not promote the level of estradiol (Lara 2002; Skarzynski and Kotwica 1993). NE also promotes follicle development (Mayerhofer 1997; Doganay 2010). Furthermore, it is revealed that NE represses VSMC proliferation through repressing ALK5 expression (Hu 2020). NE also diminishes dopaminergic cell apoptosis induced by 6-hydroxydopamine (Zhu 2019). A study conducted by Wan *et al.* (2018) revealed that Jujuboside A diminishes cardiomyocyte apoptosis induced by NE through the MAPK/AKT pathway. In this study, our data demonstrated that NE induced apoptosis in rat GCs, whereas the mechanism between NE and GC apoptosis was unclear.

From this study, we found that the level of WT1 decreased in GCs after NE treatment. And apoptosis in rat GCs induced by NE was diminished after overexpression of WT1. Our result was consistent with the study conducted by Park *et al.* (2014), which found that WT1 repressed GC apoptosis by decreasing BAX expression. WT1 level was elevated in GCs of PCOS patient (Wang 2018); it also found that WT1 played an important role in follicle development through regulating GC differentiation and the levels of E-cadherin and Par6b (Gao 2014). WT1 is needed for lineage maintenance of GCs, and inactivation of WT leads to the transformation of pre-GCs into steroidogenic cells, which leads to ovarian development defects (Cen 2020).

To further verify the regulatory mechanism of WT1 in GCs, we identified that WT1 was a putative target of miR-128-3p by dual luciferase reporter assay. miRNAs are a short, non-coding RNA which affect expression of target by inhibiting translation or degrading messenger RNA (Cheng 2019). Growing evidences revealed that miRNAs play a crucial role in the function of GCs. For example, miR-431 affects GC function via IRS2/PI3K/AKT pathway (Yang 2020). miR-21-3p suppresses GC autophagy of bovine through VEGFA/PI3K/AKT signaling (Ma 2019). In addition, a study conducted by Li *et al.* (2020) reveals that miR-146b enhanced GC apoptosis by repressing CYP19A1. And another study conducted by Zhong *et al.* (2020) revealed that miR-204-5p improves apoptosis through attenuating Bcl2 in rat ovarian granulosa. After trans-activating by steroidogenic factor 1, miR-202-5p promotes apoptosis of goat GCs through regulating TGFβR2 (Ding *et al*. 2020). And it finds that miR-30d-5p promotes GC apoptosis through suppressing Smad2 (Yu and Liu 2020). Likewise, our data demonstrated that inhibition of miR-128-3p repressed apoptosis in rat GCs induced by NE.

In summary, our finding elucidated that rno-miR-128-3p was enhanced and WT1 was alleviated in NE-treated GCs. Inhibition of rno-miR-128-3p repressed apoptosis in rat GCs induced by NE. Therefore, the miR-128-3p/WT1 pathway may serve as a potential mechanism for NE-induced GC apoptosis, and our finding may provide a new insight for the improvement of ovarian follicular development.

## Data Availability

All data generated or analyzed during this study are included in this published article.
